# Synthesis of the New Ring System Bispyrido[4',3':4,5]pyrrolo[1,2-*a*:1',2'-*d*]pyrazine and Its Deaza Analogue

**DOI:** 10.3390/molecules190913342

**Published:** 2014-08-29

**Authors:** Barbara Parrino, Virginia Spanò, Anna Carbone, Paola Barraja, Patrizia Diana, Girolamo Cirrincione, Alessandra Montalbano

**Affiliations:** Dipartimento di Scienze e Tecnologie Biologiche Chimiche e Farmaceutiche (STEBICEF), Università degli Studi di Palermo, Via Archirafi 32, 90123 Palermo, Italy

**Keywords:** diketopiperazines, plinabulin A, bispyrido-pyrrolo-pyrazine, pyrido-pyrrolo-pyrazino-indole, antiproliferative activity

## Abstract

Derivatives of the new ring systems bispyrido[4',3':4,5]pyrrolo[1,2-*a*:1',2'-*d*]pyrazine-6,13-dione and its deaza analogue pyrido[4'',3'':4',5']pyrrolo-[1',2':4,5]pyrazino[1,2-a]indole-6,13-dione were conveniently synthesized through a four-step sequence. Symmetrical derivatives of the former ring system were obtained through self condensation. On the other hand, condensation of 6-azaindole carboxylic acid with indole 2-carboxylic acid afforded the deaza analogue ring system. Derivatives of the title ring system were tested by the National Cancer Institute (Bethesda, MD, USA) and four of them exhibited modest activity against MCF7 (a breast cancer cell line) and/or UO-31 (a renal cancer cell line).

## 1. Introduction

**Figure 1:**
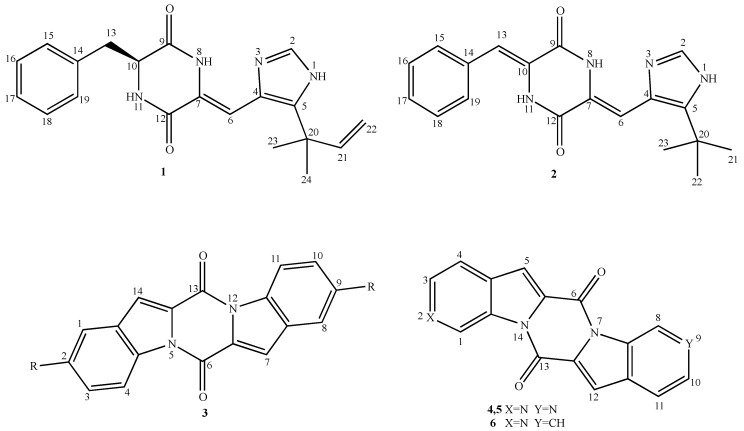
Chemical structures of diketopiperazine derivatives **1**‒**6**.

Piperazine-2,5-diones represent a very interesting class of compounds because this heterocyclic system is found in many unique natural products [1]. In recent years there has been a growing awareness of the diversity and biological roles played by many diketopiperazines among the over one-hundred found in Nature. Many derivatives have antiviral (e.g., the gliotoxins and sporidesmins), phytotoxic (e.g., cyclo(Pro-Tyr)) and antibiotic (e.g., bicyclomycin) properties, whereas other compounds show antineoplastic activity, in particular phenylahistin (**1**, Figure 1), a fungal metabolite isolated from culture broths of *Aspergillus ustus* NFC-F038, which is a result of a condensation between l-phenylalanine and an isoprenylated dehydrohistidine residue with a quaternary carbon at C-5 of the imidazole ring [2].

It is a colchicine-like microtubule binding agent endowed with cytotoxic activity against a wide variety of tumor cell lines [2,3,4], since it is able to competitively inhibit the binding site of colchicine to tubuline [3]. Phenylahistin derivatives were synthesized [5] with the aim of finding new antineoplastic derivatives, but also to understand the structural features necessary for the anti-microtubule activity. One of the most interesting compounds was revealed to be plinabulin (**2**, Figure 1) [6] a potent microtubule-targeting agent; it showed cytotoxic activity (IC_50_ = 15 nM) against human colon adenocarcinoma HT-29 cell line and it is currently in phase II clinical trials [7]. SAR studies revealed that the hydrogen bond between N8-H and N3 is crucial, allowing the formation of a rigid uniplanar pseudo-three-ring structure necessary for the binding to the microtubules.

Considering also that some properly decorated 6*H*,13*H*-pyrazino[1,2-*a*:4,5-*a'*]diindole-6,13-diones **3** that are indolo-diketopiperazines showed cytotoxic activity in the µM range against L1210 cell line [8,9,10] and, in particular, that 2,9-dimethoxy derivative gave complete inhibition of erythrocyte differentiation, whether spontaneous or induced by haemin, in leukemia K562 cell line at 50 µM, we decided to further explore the biological potential of these compounds. Considering the experience acquired in the course of our research on polycyclic nitrogen systems bearing pyrrole [11,12,13], indole [14,15,16,17,18], isoindole [19,20,21,22] and indazole [23] moieties with antitumor activity, we have decided to synthetize diaza- and aza-analogues of the ring system **3** bearing two (compounds **4**, **5**) or one (compound **6**) nitrogen atoms in the aromatic moiety in order to verify the antineoplastic properties of this new heterocyclic ring system.

Considering that the new compounds have the diketopiperazine core, capable of a colchicine-like microtubule binding, molecular docking studies were performed in order to investigate the potential binding ability of compounds **4**–**6** on tubulin. For this purpose, all compounds were docked in two different tubulin crystal structures (PDB ID code: 1SA0 [24] and 3HKD [25]) that represent two potential binding mode for colchicine site ligands.

In the 1SA0 crystal structure, colchicine, a tubulin assembly inhibitor, is the co-crystallized ligand and its binding site is located at the α,β interface of tubulin subunits [24]. In the crystal structure 3HKD, TN-16, a pyrrolidine-2,4-dione derivative, is the co-crystallized ligand. It inhibits microtubule assembly by competing with colchicine for tubulin binding [25,26]. The TN-16 binding pocket is located on the interface between the α and β subunits of the tubulin dimer and slightly extended out of the β subunit [25,27]. The X-ray crystal structures were prepared using Protein Preparation Wizard. Docking was carried out using Glide software SP mode default parameters [28].

An evaluation of the docking score results indicated that compounds **4**–**6** showed the best Glide docking score values in 3HKD (Glide score values between −9.739 and −8.927), compared to those obtained in the 1SA0 structure (Glide score values between −6.888 and −4.832) in which they did not show a good superimposition to colchicine. The only exception was for compound **4d**, that was not docked by Glide in 3HKD (Table 1).

**Table 1:** Derivatives **4a**–**d**, **5a**–**e** and **6a**–**d** docking scores for 3HKD and 1SA0.

Compound	3HKD	1SA0
**4a**	−8.927	−6.643
**4b**	−9.562	−6.675
**4c**	−9.354	−6.007
**4d**	nd	−6.455
**5a**	−9.289	−6.661
**5b**	−9.641	−6.700
**5c**	−9.203	−4.832
**5d**	−9.739	−6.477
**5e**	−9.653	−6.122
**6a**	−9.299	−6.705
**6b**	−9.648	−6.150
**6c**	−9.690	−6.380
**6d**	−9.718	−6.888

nd: Not determined.

**Figure 2:**
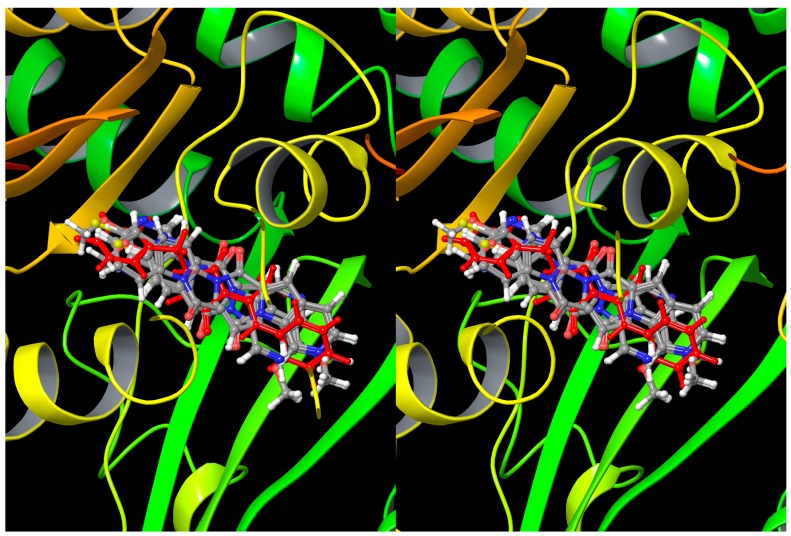
Wall eyed superimposition of compounds **4a**–**d**, **5a**–**e** and **6a**–**d** with TN-16 (red).

Analyzing the binding mode of the planned compounds in 3HKD, they showed H-bond interactions between the Glu 200 residue and one of the two carbonyl groups, interacting with the binding site in a way similar to the native ligand TN-16 (Figure 2). Although all compounds showed similar docking score values (Table 1), unsubstituted compounds **4a** and **6a** showed lower docking score values than the corresponding substituted derivatives. Generally the presence of a methoxy group in one of the two indole or aza-indole moieties seems to stabilize the tubulin-ligand complex through hydrophobic interactions with the Val 238 residue. On the basis of the docking studies we planned the synthesis of derivatives **4**–**6** in order to verify whether they were endowed with interesting biological properties.

## 2. Results and Discussion

**Scheme 1:**
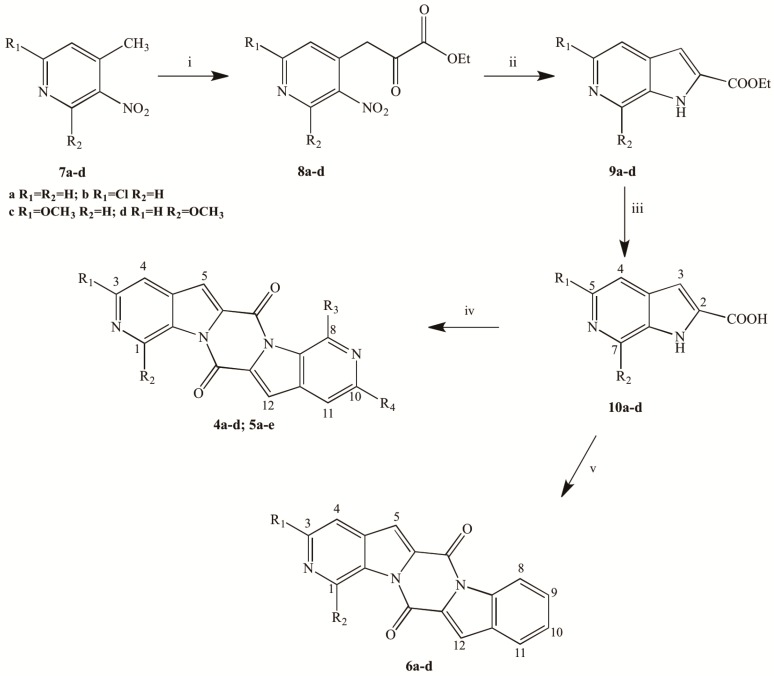
Synthesis of derivatives **4a**–**d**, **5a**–**e** and **6a**–**d**.

The key intermediates of the synthetic pathway for the pentacyclic new ring systems are 1*H*-pyrrolo[2,3-*c*]pyridine-2-carboxylic acids **10a**–**d** (Scheme 1). Commercially available pyridines **7a**,**b** were reacted with diethyl oxalate using potassium ethoxide as the base to give the corresponding derivatives **8a**,**b** in 50 and 45% yield, respectively [29]; pyridines **7c**,**d** were synthetized from the suitable 2-chloro derivatives through nucleophilic substitution with sodium methoxide [30,31]. The so-obtained methoxypyridines were reacted with diethyl oxalate using *t*-BuOK as the base allowing the isolation of compounds **8c** [30] and **8d** in good yields (72%–75%). The latter compound was isolated as the enolic tautomer. Derivatives **8a**,**b** were reduced with iron in saturated aqueous NH_4_Cl and THF to avoid halogen displacement. On the other hand compounds **8c**,**d** were dissolved in EtOH and hydrogenated over 10% Pd on charcoal. After an appropriate work-up of the reaction mixture, derivatives **9a**–**d** were obtained in good yields (60%–85%). Carboxylic acid derivatives **10a**–**d** were obtained in excellent yields (71%–95%) through alkaline hydrolysis of the corresponding ethyl esters.

Derivatives **10a**–**d** were cyclized at room temperature in anhydrous THF with 4-dimethylaminopyridine (DMAP) and 1-ethyl-3-(3-dimethylaminopropyl)carbodiimide (EDCI) as activating agents to give the new pentacyclic ring systems. Symmetrical derivatives **4a**–**d** were obtained by self-condensation of the corresponding 6-aza-indole carboxylic acids **10a**–**d** (Table 2).

*Reagents and conditions*: (i) Diethyl oxalate, potassium ethoxide in diethyl ether and EtOH, rt, 15–72 h (**8a**,**b**) or *t*-BuOK, in diethyl ether and ethanol, reflux, 4 h then 24 h rt (**8c**,**d**); (ii) Fe, saturated aqueous NH_4_Cl, THF, EtOH, reflux, 2 h (**9a**,**b**) or H_2_/Pd-C, EtOH (**9c**,**d**); (iii) NaOH 2M, EtOH, reflux 1–2 h; (iv) DMAP, EDCI, THF, rt, 48 h; (v) Indole-2-carboxylic acid, DMAP, EDCI, THF, rt, 48 h.

**Table 2:** Derivatives **4a**–**d**, **5a**–**e** and **6a**–**d**.

Compound	R_1_	R_2_	R_3_	R_4_	Yields(%)
**4a**	H	H	H	H	25
**4b**	Cl	H	H	Cl	30
**4c**	OCH_3_	H	H	OCH_3_	20
**4d**	H	OCH_3_	OCH_3_	H	28
**5a**	H	H	OCH_3_	H	40
**5b**	OCH_3_	H	OCH_3_	H	55
**5c**	H	H	H	OCH_3_	42
**5d**	Cl	H	OCH_3_	H	44
**5e**	Cl	H	H	OCH_3_	45
**6a**	H	H	-	-	33
**6b**	Cl	H	-	-	65
**6c**	OCH_3_	H	-	-	65
**6d**	H	OCH_3_	-	-	30

For the synthesis of the asymmetrical compounds **5a**–**e**, the activation of the proper acid **10a**–**d** with EDCI was followed by the addition of the suitable carboxylic acid and a further addition of EDCI in order to allow the intramolecular cyclization. In particular, **5a**–**e** were obtained from the condensation of **10a** with **10d**; **10c** with **10d**; **10a** with **10c**; **10b** with **10d**, and **10b** with **10c**, respectively (Table 2). The reaction mixture was particularly difficult to purify because of the presence not only of the asymmetrical desired derivatives **5a**–**e**, but also of 4%–6% of the symmetrical ones **4a**–**d** as byproducts of the reaction.

Moreover, through the synthetic pathway previously described it was possible to synthesize the deaza-analogues **6a**–**d** (Table 2), from the reaction between derivatives **10a**–**d** and commercially available indole-2-carboxylic acid (Scheme 1). Also in this case, not only the desired compounds **6a**–**d** were isolated from the reaction mixture, but also the symmetrical ones **4a**–**d** (3%–6%) as byproducts of the reaction together with 6*H*,13*H*-pyrazino[1,2-*a*:4,5-*a'*]diindole-6,13-dione deriving from the indole-2-carboxylic acid self-condensation (7%–9%).

All the synthesized derivatives of the new ring system 6*H*,13*H*-bispyrido[4',3':4,5]pyrrolo[1,2-*a*:1',2'-*d*]pyrazine-6,13-dione **4a**–**d**, **5a**–**e** and their deaza-analogues **6a**–**d**, were submitted to the National Cancer Institute (Bethesda, MD, USA) for screening. All derivatives were prescreened according to the NCI protocol at 10^−5^ M dose on the full panel of 60 human cancer cell lines derived from nine human cancer cell types that have been grouped in disease sub-panels including leukemia, non-small-cell lung, colon, central nervous system, melanoma, ovarian, renal, prostate and breast tumour cell lines.[32]

None of the prescreened derivatives were selected for the five dose screening (NCI-60 DTP Human Tumor Cell Line Screen), since only derivatives **5a** and **6a**, **6c** and **6d** showed moderate antineoplastic activity at micromolar concentrations. In particular derivative **5a** exhibited modest activity against the UO-31 renal cancer sub-panel cell line with a growth inhibitory percentage of 47.0; unsubstituted deaza analogue **6a** and 9-methoxy substituted derivative **6c** were shown to be selective against the MCF7 breast cancer cell line with growth inhibitory percentages of 50.6 and 39.5, respectively. More interesting results were obtained from the 11-methoxy substituted compound **6d** which was shown to be selective against both the UO-31 renal cancer sub-panel and the MCF7 breast cancer sub-panel cell lines with growth inhibitory percentages of 46.6 and 50.9, respectively.

## 3. Experimental Section

### 3.1. Chemistry

Anhydrous organic solvents were prepared by the appropriate procedures prior to use. The other organic solvents were reagent grade and used as received. Analytical TLC was performed on Merck Kieselgel 60-F254 plates. Column chromatography was performed with Merck silica gel 230–400 mesh ASTM or with a Büchi Sepacor prepacked cartridge system chromatography module.

All melting points were taken on a Buchi-Tottoli capillary apparatus and are uncorrected; IR spectra were determined in CHBr_3_, with a Shimadzu FT/IR 8400S spectrophotometer; ^1^H- and ^13^C-NMR spectra were measured in DMSO-*d*_6_ or CDCl_3_ solutions, at 200 and 50.3 MHz, respectively, using a Bruker Avance II series 200 MHz spectrometer. Elemental analyses (C, H, N) were within 0.4% of the theoretical values and were recorded with a VARIO EL III elemental analyzer.

#### 3.1.1. General Procedure for the Preparation of 2-Methoxy-pyridines **7c**,**d**

These compounds were synthesized according to the previously described procedure [30,31].

*2-Methoxy-4-methyl 5-nitropyridine* (**7c**). This compound was obtained in 95% yield. Analytical and spectroscopic data are in accordance to those reported in literature [30].

*2-Methoxy-4-methyl 3-nitropyridine* (**7d**). This compound was obtained in 80% yield. Analytical and spectroscopic data are in accordance to those reported in literature [31].

#### 3.1.2. General Procedure for the Preparation of Ethyl-3-(nitropyridin-4-yl)-2-oxopropanoates **8a**,**b**

These compounds were synthesized according to the previously described procedure [29].

*Ethyl-3-(3-nitropyridin-4-yl)-2-oxopropanoate* (**8a**). This compound was obtained in 50% yield. Analytical and spectroscopic data are in accordance to those reported in literature [29].

*Ethyl-3-(2-chloro-5-nitropyridin-4-yl)-2-oxopropanoate* (**8b**). This compound was obtained in 45% yield. Analytical and spectroscopic data are in accordance to those reported in literature [29].

#### 3.1.3. General Procedure for the Preparation of Ethyl-3-(nitropyridin-4-yl)-2-oxopropanoates **8c**,**d**

To a stirred solution of *t*-BuOK (2.4 mmol) in anhydrous EtOH (1 mL) and diethyl ether (10 mL) diethyl oxalate (2.4 mmol, 0.3 mL) was added under a nitrogen atmosphere. The reaction mixture was kept at room temperature for 15 min, then a solution of the suitable pyridine **7c**,**d** (2.4 mmol) was added and the reaction mixture was refluxed for 4 h and stirred at room temperature 24 h. The orange residue thus obtained was shaken in diethyl ether, filtered off and air dried. Water (9.2 mL) was added and acetic acid was added until pH 4.0. The desired product was filtered off, and dried in the desiccator to afford the desired products as cream solids.

*Ethyl 3-(2-methoxy-5-nitropyridin-4-yl)-2-oxopropanoate* (**8c**). This compound was obtained in 72% yield. Analytical and spectroscopic data are in accordance to those reported in literature [30,33].

*Ethyl 3-(2-methoxy-3-nitropyridin-4-yl)-2-oxopropanoate* (**8d**). Title compound **8d** was isolated as the enolic tautomer. Rf = 0.33 (CH_2_Cl_2_); mp 78.4–79.6 °C; yield 75%; IR: 3426 (OH), 1706 (CO) cm^1^; ^1^H-NMR (DMSO-*d*_6_) δ: 1.28 (3H, t, *J =* 6.0 Hz, CH_3_), 3.97 (3H, s, OCH_3_), 4.28 (2H, q, *J =* 6.0 Hz, CH_2_), 6.00 (1H, s, CH), 7.86 (1H, d, *J =* 6.00 Hz, H-5), 8.34 (1H, d, *J =* 6.0 Hz, H-6), 11.30 (1H, bs, OH). ^13^C-NMR (DMSO-*d*_6_) δ: 13.9 (q), 54.4 (q), 62.2 (t), 97.2 (d), 116.4 (d), 132.7 (s), 136.3 (s), 148.3 (d), 148.8 (s), 154.3 (s), 163.0 (s). Anal. Calcd for C_11_H_12_N_2_O_6_ (268.22): C, 49.26; H, 4.51; N, 10.44. Found: C, 49.21; H, 4.75; N, 10.16.

#### 3.1.4. General Procedure for the Preparation of Ethyl 1*H*-pyrrolo[2,3-*c*]pyridine-2-carboxylates **9a**,**b**

These compounds were synthesized according to the previously described procedure [29,34].

*Ethyl 1H-pyrrolo[2,3-c]pyridine-2-carboxylate* (**9a**). This compound was obtained in 60% yield. Analytical and spectroscopic data are in accordance to those reported in literature [29].

*Ethyl 5-chloro-1H-pyrrolo[2,3-c]pyridine-2-carboxylate* (**9b**). This compound was obtained in 60% yield. Analytical and spectroscopic data are in accordance to those reported in literature [34].

#### 3.1.5. General Procedure for the Preparation of Ethyl 1*H*-pyrrolo[2,3-*c*]pyridine-2-carboxylates **9c**,**d**

Derivatives **8c**,**d** (2.9 mmol) were dissolved in EtOH (40 mL) and hydrogenated over 10% Pd on charcoal. The catalyst was removed by filtration under argon and the solvent was evaporated *in vacuo*.

*Ethyl 5-methoxy-1H-pyrrolo[2,3-c]pyridine-2-carboxylate* (**9c**). This compound was obtained in 85% yield. Analytical and spectroscopic data are in accordance to those reported in literature [33].

*Ethyl 7-methoxy-1H-pyrrolo[2,3-c]pyridine-2-carboxylate* (**9d**). Title compound **9d** was purified by flash-chromatography using CH_2_Cl_2_/ethyl acetate 96:4. Rf = 0.63 (CH_2_Cl_2_/ethyl acetate 95:5) as a white powder; mp 134.1–135.0 °C; yield 75%; IR: 3435 (NH), 1708 (CO) cm^1^; ^1^H-NMR (CDCl_3_) δ: 1.41 (3H, t, *J =* 6.0 Hz, CH_3_), 4.09 (3H, s, OCH_3_), 4.43 (2H, q, *J =* 6.0 Hz, CH_2_), 7.13–7.17 (2H, m, H-3, H-4), 7.77 (1H, d, *J =* 6.0 Hz, H-5), 9.61 (1H, bs, NH). ^13^C-NMR (CDCl_3_) δ: 14.3 (q), 53.3 (q), 61.4 (t), 107.7 (d), 110.8 (d), 122.3 (s), 129.3 (s), 133.0 (s), 135.9 (d), 151.9 (s), 161.4 (s). Anal. Calcd for C_11_H_12_N_2_O_3_ (220.22): C, 59.99; H, 5.49; N, 12.72. Found: C, 60.14; H, 5.66; N, 12.57.

#### 3.1.6. General Procedure for the Preparation of 1*H*-pyrrolo[2,3-*c*]pyridine-2-carboxylic Acids **10a**–**d**

To a stirred solution of **9a**–**d** (1.3 mmol) in EtOH (12 mL) 2M NaOH was added (1.7 mmol, 1.1 mL). The reaction mixture was heated under reflux for 1h (**10a**) or 2h (**10b**) and the solvent was evaporated. Water (10 mL) was added and acetic acid was added until pH 4.0. The desired product was filtered off, dried into the desiccators to afford the desired product.

*1H-Pyrrolo[2,3-c]pyridine-2-carboxylic acid* (**10a**). This compound was obtained in 95% yield. Analytical and spectroscopic data are in accordance to those reported in literature [29].

*5-Chloro-1H-pyrrolo[2,3-c]pyridine-2-carboxylic acid* (**10b**). This compound was obtained in 71% yield. Analytical and spectroscopic data are in accordance to those reported in literature [29].

*5-Methoxy-1H-pyrrolo[2,3-c]pyridine-2-carboxylic acid* (**10c**). This compound was obtained in 80% yield. Analytical and spectroscopic data are in accordance to those reported in literature [35].

*7-Methoxy-1H-pyrrolo[2,3-c]pyridine-2-carboxylic acid* (**10d**). This compound was obtained after 1 h reflux as a white powder. Rf *=* 0.40 (CH_2_Cl_2_/MeOH 9:1); mp 269.3–271.1 °C; yield 82%; IR: 3550 (NH), 3311 (OH), 1718 (CO) cm^−1^; ^1^H-NMR (DMSO-*d*_6_) δ: 4.02 (3H, s, OCH_3_), 7.07 (1H, s, H-3), 7.21 (1H, d, *J =* 6.0 Hz , H-4), 7.68 (1H, d, *J =* 6.0 Hz, H-5), 9.61(1H, bs, NH), 12.30 (1H, bs, OH). ^13^C-NMR (DMSO-*d*_6_) δ: 52.7 (q), 106.8 (d), 110.5 (d), 122.0 (s), 131.5 (s), 132.6 (s), 134.8 (d), 151.6 (s), 162.3 (s). Anal. Calcd for C_9_H_8_N_2_O_3_(192.17): C, 56.25; H, 4.20; N, 14.58. Found: C, 56.29; H, 4.24; N, 14.37.

#### 3.1.7. General Procedure for the Preparation of 6*H*,13*H*-Bispyrido[4',3':4,5]pyrrolo[1,2-*a*:1',2'-*d*]pyrazine-6,13-diones **4a**–**d**

To a stirred solution of **10a**–**d** (2.3 mmol) in anhydrous THF (20 mL) dimethylaminopyridine (DMAP, 2.3 mmol) was added, followed by EDCI (4.8 mmol) addition after 10 min; the reaction mixture was stirred for 48h at room temperature. The solid was collected by filtration and recrystallizated from CH_2_Cl_2_ and MeOH, affording the desired products as yellow solids. Compounds **4a**–**d** were characterized only by ^1^H-NMR spectroscopy. The poor solubility of the title compounds prevented the ^13^C-NMR spectra from being recorded.

*6H,13H-Bispyrido[4',3':4,5]pyrrolo[1,2-a:1',2'-d]pyrazine-6,13-dione* (**4a**). Rf *=* 0.34 (CH_2_Cl_2_/MeOH 95:5); mp 352.3–353.9 °C; yield 25%; IR: 1722 (CO) cm^1^; ^1^H-NMR (DMSO-*d*_6_) δ: 7.91 (2H, d, *J =* 6.0 Hz, H-4 and H-11), 7.92 (2H, s, H-5 and H-12), 8.60 (2H, d, *J =* 6.0 Hz, H-3 and H-10), 9.71 (2H, s, H-1 and H-8). Anal. Calcd for C_16_H_8_N_4_O_2_ (288.26): C, 66.67; H, 2.80; N, 19.44. Found: C, 66.62; H, 2.84; N, 19.39.

*3,10-Dichloro-6H,13H-bispyrido[4',3':4,5]pyrrolo[1,2-a:1',2'-d]pyrazine-6,13-dione* (**4b**). Rf *=* 0.60 (CH_2_Cl_2_/MeOH 98:2); mp 380.6–381.9 °C; yield 30%; IR: 1716 (CO) cm^1^; ^1^H-NMR (DMSO-*d*_6_) δ: 7.88 (2H, s, H-4 and H-11), 8.05 (2H, s, H-5 and H-12), 9.47 (2H, s, H-1 and H-8). Anal. Calcd for C_16_H_6_Cl_2_N_4_O_2_ (357.15): C, 53.81; H, 1.69; N, 15.69. Found: C, 53.89; H, 1.78; N, 15.97.

*3,10-Dimethoxy-6H,13H-bispyrido[4',3':4,5]pyrrolo[1,2-a:1',2'-d]pyrazine-6,13-dione* (**4c**). Rf *=* 0.57 (CH_2_Cl_2_/MeOH 98:2); mp 343.0–344.2 °C; yield 20%; IR: 1710 (CO) cm^−1^; ^1^H-NMR (DMSO-*d*_6_) δ: 3.95 (3H, s, OCH_3_), 7.26 (2H, s, H-4 and H-11), 7.73 (2H, s, H-5 and H-12), 9.27 (2H, s, H-1 and H-8). Anal. Calcd for C_18_H_12_N_4_O_4_ (348.31): C, 62.07; H, 3.47; N, 16.09. Found: C, 61.92; H, 3.53; N, 15.95.

*1,8-Dimethoxy-6H,13H-bispyrido[4',3':4,5]pyrrolo[1,2-a:1',2'-d]pyrazine-6,13-dione* (**4d**). Rf *=* 0.45 (CH_2_Cl_2_/MeOH 98:2); mp 380.6–381.9 °C; yield 28%; IR: 1723 (CO) cm^−1^; ^1^H-NMR (DMSO-*d*_6_) δ: 4.06 (3H, s, OCH_3_), 7.44 (2H, d, *J =* 6.0 Hz, H-4 and H-11), 7.79 (2H, s, H-5 and H-12), 8.12 (2H, d, *J =* 6.0 Hz, H-3 and H-10). Anal. Calcd for C_18_H_12_N_4_O_4_ (348.31): C, 62.07; H, 3.47; N, 16.09. Found: C, 61.83; H, 3.66; N, 16.05.

#### 3.1.8. General Procedure for the Preparation of 6*H*,13*H*-bispyrido[4',3':4,5]pyrrolo[1,2-*a*:1',2'-*d*]pyrazine-6,13-diones **5a**–**e**

To a stirred solution of **10a**–**d** (2.3 mmol) in anhydrous THF (20 mL) dimethylaminopyridine (DMAP, 2.3 mmol) was added, followed by EDCI (1.2 mmol) after 10 min; the reaction mixture was stirred at room temperature for 1h. The suitable acid **10a**–**d** (1.0 mmol) and EDCI (1.2 mmol) were added and the reaction mixture was stirred for 48 h. The solid was collected by filtration, purified by flash chromatography using CH_2_Cl_2_/MeOH 98:2 and recrystallized from CH_2_Cl_2_ and MeOH, affording the desired product as a yellow solid. Compounds **5a**–**e** were characterized only by ^1^H-NMR spectroscopy. The poor solubility of the title compounds prevented ^13^C-NMR spectra from being recorded.

*8-Methoxy-6H,13H-bispyrido[4',3':4,5]pyrrolo[1,2-a:1',2'-d]pyrazine-6,13-dione* (**5a**). This product was obtained by reaction of **10a** with **10d**. Rf *=* 0.46 (CH_2_Cl_2_/MeOH 98:2); mp 328.4–329.0 °C; yield 40%; IR: 1712 (CO), 1694 (CO) cm^1^; ^1^H-NMR (DMSO-*d*_6_) δ: 4.06 (3H, s, OCH_3_), 7.45 (1H, d, *J =* 6.0 Hz, H-11), 7.82 (1H, s, H-12), 7.89–7.92 (2H, m, H-5 and H-4), 8.14 (1H, d, *J =* 6.0 Hz, H-10), 8.60 (1H, d, *J =* 4.0 Hz, H-3), 9.67 (1H, s H-1). Anal. Calcd for C_17_H_10_N_4_O_3_ (318.29): C, 64.15; H, 3.17; N, 17.60. Found: C, 63.87; H, 3.13; N, 17.75. From this reaction derivatives **4a** (yield 4%) and **4d** (yield 6%) were also isolated.

*1,10-Dimethoxy-6H,13H-bispyrido[4',3':4,5]pyrrolo[1,2-a:1',2'-d]pyrazine-6,13-dione* (**5b**). This product was obtained by reaction of **10c** with **10d**. Rf *=* 0.34 (CH_2_Cl_2_/MeOH 95:5); mp 309.1–309.4 °C; yield 55%; IR: 1712 (CO), 1689 (CO) cm^−1^; ^1^H-NMR (DMSO-*d*_6_) δ: 3.94 (3H, s, OCH_3_), 4.05 (3H, s, OCH_3_), 7.25 (1H, s, H-12), 7.42 (1H, d, *J =* 4.0 Hz, H-4), 7.79 (1H, s, H-11), 7.84 (1H, s, H-5), 8.12 (1H, d, *J =* 4.0 Hz, H-3), 9.24 (1H, s, H-8). Anal. Calcd for C_18_H_12_N_4_O_4_ (348.31): C, 62.07; H, 3.47; N, 16.09. Found: C, 62.20; H, 3.42; N, 16.25. From this reaction derivatives **4c** (yield 5%) and **4d** (yield 6%) were also isolated.

*3-Methoxy-6H,13H-bispyrido[4',3':4,5]pyrrolo[1,2-a:1',2'-d]pyrazine-6,13-dione* (**5c**). This product was obtained by reaction of **10a** with **10c**. Rf *=* 0.37 (CH_2_Cl_2_/MeOH 98:2); mp 271.1–271.8 °C; yield 42%; IR: 1718 (CO), 1707 (CO) cm^−1^; ^1^H-NMR (DMSO-*d*_6_) δ: 3.95 (3H, s, OCH_3_), 7.26 (1H, s, H-12), 7.78 (1H, s, H-5), 7.87 (1H, s, H-4), 7.90 (1H, d, *J =* 6.0 Hz, H-11), 7.59 (1H, d, *J =* 6.0 Hz, H-10), 9.28 (1H, s, H-8), 9.68 (1H, s, H-1). Anal. Calcd for C_17_H_10_N_4_O_3_ (318.29): C, 64.15; H, 3.17; N, 17.60. Found: C, 64.06; H, 3.08; N, 17.89. From this reaction derivatives **4a** (yield 4%) and **4c** (yield 5%) were also isolated.

*10-Chloro-1-methoxy-6H,13H-bispyrido[4',3':4,5]pyrrolo-[1,2-a:1',2'-d]pyrazine-6,13-dione* (**5d**). This product was obtained by reaction of **10b** with **10d**. Rf *=* 0.47 (CH_2_Cl_2_/MeOH 98:2); mp 292.2–293.0 °C; yield 44%; IR: 1712 (CO), 1690 (CO) cm^−1^; ^1^H-NMR (DMSO-*d*_6_) δ: 4.06 (3H, s, OCH_3_), 7.45 (1H, d *J =* 6.0 Hz, H-4), 7.75 (1H, s, H-5), 7.92 (1H, s, H-12), 8.05 (1H, s, H-11), 8.14 (1H, d, *J =* 6.0 Hz, H-3), 9.44 (1H, s, H-8). Anal. Calcd for C_17_H_9_ClN_4_O_3_ (352.73): C, 57.89; H, 2.57; N, 15.88. Found: C, 57.60; H, 2.48; N, 15.96. From this reaction derivatives **4b** (yield 6%) and **4d** (yield 5%) were also isolated.

*3-Chloro-10-methoxy-6H,13H-bispyrido[4',3':4,5]-pyrrolo[1,2-a:1',2'-d]pyrazine-6,13-dione* (**5e**). This product was obtained by reaction of **10b** with **10c**. Rf *=* 0.56 (CH_2_Cl_2_/MeOH 98:2); mp 312.0–312.5 °C; yield 45%; IR: 1720 (CO), 1705 (CO) cm^−1^; ^1^H-NMR (DMSO-*d*_6_) δ: 3.96 (3H, s, OCH_3_), 7.26 (1H, s, H-11), 7.80 (1H, s, H-12), 7.82 (1H, s, H-5), 8.04 (1H, s, H-4), 9.27 (1H, s, H-8), 9.46 (1H, s, H-1). Anal. Calcd for C_17_H_9_ClN_4_O_3_ (352.73): C, 57.89; H, 2.57; N, 15.88. Found: C, 57.80; H, 2.49; N, 16.16. From this reaction derivatives **4b** (yield 5%) and **4c** (yield 5%) were also isolated.

#### 3.1.9. General Procedure for the Preparation of 6*H*,13*H*-Pyrido[4'',3'':4',5']pyrrolo[1',2':4,5]pyrazino[1,2-*a*]indole-6,13-diones **6a**–**d**

To a stirred solution of the suitable **10a**–**d** (1.2 mmol) in anhydrous THF (20 mL) dimethylaminopyridine (DMAP) (1.2 mmol) was added, followed by EDCI (1.2 mmol) after 10 min; the reaction mixture was stirred at room temperature for 1h. Indole 2-carboxylic acid (1.0 mmol) and EDCI (1.2 mmol) were added and the reaction mixture was stirred for 48 h. The solid was collected by filtration, purified by flash chromatography using using CH_2_Cl_2_/MeOH 98:2 and recrystallized with CH_2_Cl_2_ and MeOH, affording the desired products as yellow solid. Compounds **6a**–**d** were characterized only by ^1^H-NMR spectroscopy. The poor solubility of the title compounds prevented ^13^C-NMR spectra from being recorded.

*6H,13H-Pyrido[4'',3'':4',5']pyrrolo[1',2':4,5]pyrazino[1,2-a]indole-6,13-dione* (**6a**). Rf *=* 0.28 (CH_2_Cl_2_/MeOH 98:2); mp 347.4–347.8 °C; yield 33%; IR: 1701 (broad, CO) cm^−1^;^1^H-NMR (DMSO-*d*_6_) δ: 7.48 (1H, td, *J =* 6.0 2.0 Hz, H-9), 7.67 (1H, td, *J =* 6.0 2.0 Hz, H-10), 7.85 (1H, s, H-12), 7.88–7.93 (3H, m, H-4, H-5 and H-8), 8.48 (1H, d, *J =* 6.0 Hz, H-11), 8.58 (1H, d, *J =* 6.0 Hz, H-3), 9.71 (1H, s, H-1). Anal. Calcd for C_17_H_9_N_3_O_2_ (287.27): C, 71.08; H, 3.16; N, 14.63. Found: C, 71.29; H, 3.29; N, 14.84. From this reaction derivatives **4a** (yield 5%) and 6*H*,13*H*-pyrazino[1,2-*a*:4,5-*a'*]diindole-6,13-dione (yield 8%) whose analytical and spectroscopic data are in accordance to those reported in literature were also isolated [36].

*3-Chloro-6H,13H-pyrido[4'',3'':4',5']pyrrolo[1',2':4,5]pyrazino[1,2-a]indole-6,13-dione* (**6b**). Rf *=* 0.63 (CH_2_Cl_2_/MeOH 98:2); mp 306.3–306.7 °C; yield 65%; IR: 1700 (broad, CO) cm^−1^; ^1^H-NMR (DMSO-*d*_6_) δ: 7.49 (1H, t, *J =* 8.0 Hz, H-9), 7.67 (1H, t, *J =* 8.0 Hz, H-10), 7.77 (1H, s, H-12), 7.92 (1H, d, *J =* 8.0 Hz, H-8), 7.95 (1H, s, H-5), 8.02 (1H, s, H-4), 8.48 (1H, d, *J =* 8.0 Hz, H-11), 9.48 (1H, s, H-1). Anal. Calcd for C_17_H_8_ClN_3_O_2_ (321.72): C, 63.47; H, 2.51; N, 13.06. Found: C, 63.68; H, 2.46; N, 13.30. From this reaction were also isolated derivatives **4b** (yield 3%) and 6*H*,13*H*-pyrazino[1,2-*a*:4,5-*a'*]diindole-6,13-dione (yield 7%) whose analytical and spectroscopic data are in accordance to those reported in literature [36].

*3-Methoxy-6H,13H-pyrido[4'',3'':4',5']pyrrolo[1',2':4,5]pyrazino[1,2-a]indole-6,13-dione* (**6c**). Rf *=* 0.65 (CH_2_Cl_2_/MeOH 98:2); mp 279.0–279.4 °C; yield 65%; IR: 1727 (CO), 1702 (CO) cm^−1^; ^1^H-NMR (DMSO-*d*_6_) δ: 3.95 (3H, s, OCH_3_), 7.24 (1H, s, H-4), 7.47 (1H, t, *J =* 8.0 Hz, H-9), 7.65 (1H, t, *J =* 8.0 Hz, H-10), 7.71 (1H, s, H-12), 7.87 (1H, s, H-5), 7.91 (1H, d, *J =* 8.0 Hz, H-8), 8.46 (1H, d, *J =* 8.0 Hz, H-11), 9.29 (1H, s, H-1). Anal. Calcd for C_18_H_11_N_3_O_3_ (317.30): C, 68.14; H, 3.49; N, 13.24. Found: C, 68.09; H, 3.70; N, 13.13. From this reaction were also isolated derivatives **4c** (yield 4%) and 6*H*,13*H*-pyrazino[1,2-*a*:4,5-*a'*]diindole-6,13-dione (yield 7%) whose analytical and spectroscopic data are in accordance to those reported in literature [36].

*1-Methoxy-6H,13H-pyrido[4'',3'':4',5']pyrrolo[1',2':4,5]pyrazino[1,2-a]indole-6,13-dione* (**6d**). Rf *=* 0.63 (CH_2_Cl_2_/MeOH 98:2); mp 283.8–283.9 °C; yield 30%; IR: 1712 (CO), 1690 (CO) cm^−1^; ^1^H-NMR (DMSO-*d*_6_) δ: 4.05 (3H, s, OCH_3_), 7.41–7.50 (2H, m, H-4 and H-9), 7.64 (1H, t, *J =* 8.0 Hz, H-10), 7.81 (2H, s, H-5 and H-12), 7.90 (1H, d, *J =* 8.0 Hz, H-8), 8.10 (1H, d, *J =* 6.0 Hz, H-3), 8.43 (1H, d, *J =* 8.0 Hz, H-11). Anal. Calcd for C_18_H_11_N_3_O_3_ (317.30): C, 68.14; H, 3.49; N, 13.24. Found: C, 68.39; H, 3.45; N, 12.95. From this reaction were also isolated derivatives **4d** (yield 6%) and 6*H*,13*H*-pyrazino[1,2-*a*:4,5-*a'*]diindole-6,13-dione (yield 9%) whose analytical and spectroscopic data are in accordance to those reported in literature [36].

### 3.2. Docking

Docking studies were performed for all designed compounds by Glide 5.9 (Schrödinger Inc., New York, NY, USA, 2013). The X-ray crystallographic structures of tubulin (PDB code 3HKD [24] and 1SA0 [23]) were downloaded from Protein Data Bank [37]. For Glide docking studies, the stathmin-like domain and chains B, C were removed. The proteins were minimized by Protein Preparation Wizard. Partial atomic charges were assigned according to the OPLS_2005 force field. A radius of 20 Å was selected for active site cavity during receptor grid generation with the center defined by the co-crystallized ligand TN-16 and colchicine. All compounds used in the docking study with Glide were built within Maestro by using the build module of Schrödinger Inc. (2013). Docking calculations were performed using standard mode of Glide Program. To validate the Glide docking protocol, TN-16 was redocked into the binding site. The docking structure was compared to the crystal structure showing that this protocol successfully reproduces the crystal TN-16 tubulin complex.

### 3.3. Biology

#### Methodology of the *in Vitro* Cancer Screen

*In vitro* cancer screens were done according to the NCI protocol at 10^−5^ M dose on the full panel of 60 human cancer cell lines derived from nine human cancer cell types that have been grouped in disease sub-panels including leukemia (CCRF-CEM, HL-60(TB), K-562, MOLT-4, RPMI-8226, SR), non-small-cell lung (A549/ATCC, EKVX, HOP-62, HOP-92, NCI-H226, NCI-H23, NCI-H322M, NCI-H460, NCI-H522), colon (COLO 205, HCC-2998, HCT-116, HCT-15, HT29, KM12, SW-620), central nervous system (SF-268, SF-295, SF-539, SNB-19, SNB-75, U251), melanoma (LOX IMVI, MALME-3M, M14, MDA-MB-435, SK-MEL-2, SK-MEL-28, SK-MEL-5, UACC-257, UACC-62), ovarian (IGROV1, OVCAR-3, OVCAR-4, OVCAR-5, OVCAR-8, NCI/ADR-RES, SK-OV-3), renal (786-0, A498, ACHN, CAKI-1, RXF 393, SN12C, TK-10, UO-31), prostate (PC-3, DU-145) and breast tumour (MCF7, MDA-MB-231/ATCC, HS 578T, BT-549, T-47D, MDA-MB-468) cell lines [32].

The human tumor cell lines of the cancer screening panel are grown in RPMI 1640 medium containing 5% fetal bovine serum and 2 mM l-glutamine. For a typical screening experiment, cells are inoculated into 96 well microtiter plates in 100 µL at plating densities ranging from 5000 to 40,000 cells/well depending on the doubling time of individual cell lines. After cell inoculation, the microtiter plates are incubated at 37 °C, 5% CO_2_, 95% air and 100% relative humidity for 24 h prior to addition of experimental drugs. After 24 h, two plates of each cell line are fixed *in situ* with TCA, to represent a measurement of the cell population for each cell line at the time of drug addition (Tz). Experimental drugs are solubilized in dimethyl sulfoxide at 400-fold the desired final maximum test concentration and stored frozen prior to use. At the time of drug addition, an aliquot of frozen concentrate is thawed and diluted to twice the desired final maximum test concentration with complete medium containing 50 µg/mL gentamicin. Aliquots of 100 µL of drug are added to the appropriate microtiter wells already containing 100 µL of medium, resulting in the required final drug concentration. Following drug addition, the plates are incubated for an additional 48 h at 37 °C, 5% CO_2_, 95% air, and 100% relative humidity. For adherent cells, the assay is terminated by the addition of cold TCA. Cells are fixed *in situ* by the gentle addition of 50 µL of cold 50% (w/v) TCA (final concentration, 10% TCA) and incubated for 60 min at 4 °C. The supernatant is discarded, and the plates are washed five times with tap water and air dried. Sulforhodamine B (SRB) solution (100 µL) at 0.4% (w/v) in 1% acetic acid is added to each well, and plates are incubated for 10 min at room temperature. After staining, unbound dye is removed by washing five times with 1% acetic acid and the plates are air dried. Bound stain is subsequently solubilized with 10 mM trizma base, and the absorbance is read on an automated plate reader at a wavelength of 515 nm. For suspension cells, the methodology is the same except that the assay is terminated by fixing settled cells at the bottom of the wells by gently adding 50 µL of 80% TCA (final concentration, 16% TCA). Using the seven absorbance measurements [time zero, (Tz), control growth, (C), and test growth in the presence of drug (Ti)], the percentage growth is calculated. Percentage growth inhibition is calculated as:

*[(Ti - Tz)/(C − Tz)] × 100* for concentrations for which Ti ≥ Tz (1)

*[(Ti - Tz)/Tz] × 100* for concentrations for which Ti ˂ Tz (2)

For further information to see NCI website [38].

## 4. Conclusions

In conclusion, we have reported the synthesis of derivatives of the new ring systems 6*H*,13*H*-bispyrido[4',3':4,5]pyrrolo[1,2-*a*:1',2'-*d*]pyrazine-6,13-dione **4**, **5** and 6*H*,13*H*-pyrido[4'',3'':4',5']-pyrrolo[1',2':4,5]pyrazino[1,2-*a*]indole-6,13-dione **6** using a simple and versatile synthetic pathway. All derivatives were prescreened according to the NCI protocol at 10^−5^ M dose on the full panel of 60 human cancer cell lines derived from nine human cancer cell types. Only derivatives **5a** and **6a**, **6c** and **6d** showed a moderate antineoplastic activity at micromolar concentration. In particular derivative **5a** exhibited modest activity against the UO-31 renal cancer sub-panel cell line; deaza analogue **6a** and the 9-methoxy substituted derivative **6c** were shown to be selective against the MCF7 breast cancer cell line. More interesting results were obtained from the 11-methoxy substituted compound **6d** which showed selectivity against both the UO-31 renal cancer sub-panel and the MCF7 breast cancer sub-panel cell lines. Unfortunately the moderate activity showed by derivatives **5a** and **6a**, **6c** and **6d** against a limited number of cell lines could not allow a reliable SAR evaluation. However, the antiproliferative activity shown by derivatives **5a** and **6a**, **6c** and **6d**, although modest, encourages further studies directed toward the synthesis of new compounds with an improved growth inhibitory effect.
